# Vitamin D Deficiency in Mexican Pregnant Women: Is Supplementation with ≤400 IU/day Enough?

**DOI:** 10.3390/nu12092517

**Published:** 2020-08-20

**Authors:** Otilia Perichart-Perera, Carla Patricia González-Leyva, Isabel González-Ludlow, Maricruz Tolentino-Dolores, Mario Solis-Paredes, Enrique Reyes-Muñoz, Hector Borboa-Olivares, Maribel Sánchez-Martínez, Sandra Parra-Hernández, Eric Monterrubio-Flores, Lourdes Schnaas y Arrieta, Mario Guzmán-Huerta, Guadalupe Estrada-Gutierrez

**Affiliations:** 1 Departamento de Nutrición y Bioprogramación, Instituto Nacional de Perinatología, Ciudad de México 11000, Mexico; otiliaperichart@inper.gob.mx (O.P.-P.); carlapaty90@hotmail.com (C.P.G.-L.); isaglezludlow@icloud.com (I.G.-L.); cruz_tolentino@yahoo.com.mx (M.T.-D.); 2 Departamento de Genética y Genómica Humana, Instituto Nacional de Perinatología, Ciudad de México 11000, Mexico; msolis_83@yahoo.com.mx; 3 Coordinación de Endocrinología Ginecológica y Perinatal, Instituto Nacional de Perinatología, Ciudad de México 11000, Mexico; dr.enriquereyes@gmail.com; 4 Subdirección de Investigación en Intervenciones Comunitarias, Instituto Nacional de Perinatología, Ciudad de México 11000, Mexico; h_borboa1@yahoo.com; 5 Departamento de Inmunobioquímica, Instituto Nacional de Perinatología, Ciudad de México 11000, Mexico; maribel71sm@yahoo.com.mx (M.S.-M.); rebe1602@hotmail.com (S.P.-H.); 6 Centro de Investigación en Nutrición y Salud, Instituto Nacional de Salud Pública, Cuernavaca 62100, Mexico; eric@insp.mx; 7 Departamento de Neurobiología del Desarrollo, Instituto Nacional de Perinatología, Ciudad de México 11000, Mexico; lschnaas@hotmail.com; 8 Departamento de Medicina Traslacional, Instituto Nacional de Perinatología, Ciudad de México 11000, Mexico; mguzmanhuerta@yahoo.com.mx; 9 Dirección de Investigación, Instituto Nacional de Perinatología, Ciudad de México 11000, Mexico

**Keywords:** pregnancy, serum 25-OH-D, vitamin D3, vitamin D status

## Abstract

Controversy remains surrounding vitamin D routine supplementation in healthy pregnancy, and the doses are unclear. The aim of this study was to describe maternal vitamin D status throughout pregnancy in a group of Mexican women and evaluate the effect of frequently prescribed doses of vitamin D3 on longitudinal 25-OH-D concentrations, adjusting for obesity, season, and other factors. We conducted a cohort study (Instituto Nacional de Perinatología-INPer) (2017–2020)) of healthy pregnant women without complications. Pregestational overweight/obesity (body mass index ≥ 25), vitamin D3 supplementation (prescribed by physician; 0–250, 250–400, and >400 IU/day), and serum 25-OH-D concentrations (ELISA) were evaluated in each trimester of pregnancy. Vitamin D deficiency or insufficiency was computed (<20 and <30 ng/mL, respectively). We studied 141 adult women; 58.5% had pregestational obesity or overweight. In the first trimester, 45.8% of the women were supplemented with vitamin D3; 51.4% had vitamin D insufficiency and 37.3%, deficiency. In the third trimester, 75.4% of the women were supplemented, and 20% of them still had deficiency. The final general mixed linear model showed that 25-OH-D significantly increased throughout pregnancy (*p* < 0.001); the highest increase was observed in the third trimester in women with doses >400 IU/day of vitamin D3 (+4 ng/mL, 95% CI: 1.72–8.11 ng/mL). In winter/autumn, 25-OH-D concentrations were also lower (*p* ≤ 0.05). In this group of pregnant Mexican women, the prevalence of vitamin D deficiency and insufficiency was high. A higher increase in 25-OH-D concentrations during pregnancy was observed when the women were supplemented with >400 IU/day. Common supplementation doses of 250–400 IU/day were insufficient for achieving an adequate maternal vitamin D status.

## 1. Introduction

Nutrition plays a central role in promoting adequate fetal growth and supporting the physiological and metabolic changes that occur during pregnancy. It is well established that healthy eating and adequate nutrient supplementation result in optimal nutrition and metabolic fetal programming, associated with a decreased risk of obesity and diabetes mellitus later in life [1,2,3].

Vitamin D is a liposoluble vitamin and hormone that is synthesized in the skin, and it is normally related with bone health. Vitamin D also regulates multiple body functions, playing important immunologic and anti-inflammatory roles [4,5].

Maternal vitamin D deficiency (25-hydroxivitamin D (25-OH-D) < 20 ng/mL) represents a public health problem and is considered the least diagnosed and treated nutrition deficiency around the world [4]. According to a global report, vitamin D deficiency during pregnancy was present in 42–72% of women from the Americas, 18–90% of those from Europe, and 46% of those from the Eastern Mediterranean. However, data from the Americas region were derived only from studies in the U.S. and Canada [6]. A recent cohort study in Brazil reported 21% of pregnant women having vitamin D deficiency [7]. In Mexico, one study reported that 37% of women of reproductive age had vitamin D deficiency, and 50% had insufficiency (<30 ng/mL) [8]. In a study of Mexican mother–child pairs, 61% of pregnant women and 98% of newborns presented with vitamin D deficiency [9].

In pregnant women with low concentrations of 25-OH-D, significantly higher risks of developing gestational diabetes mellitus (GDM), preeclampsia, preterm birth, and delivering a small-for-gestational-age newborn have been observed, among other complications [10,11]. Maternal vitamin D status may be affected by multiple factors such as season of the year, sun exposure, dietary intake, and, in some populations, obesity [12]. Maternal vitamin D supplementation was reported to be the strongest predictor of 25-OH-D tracking during pregnancy [13].

Even though several studies have indicated that maternal vitamin D deficiency increases the risk of adverse perinatal outcomes, controversy exists regarding routine maternal supplementation and the amount that should be recommended. The World Health Organization (WHO) does not recommend routine vitamin D supplementation during pregnancy, except in documented deficiency status, where doses should be aligned with dietary intake recommendations [2]. However, dietary intake recommendations around the world are inconsistent. The WHO dietary intake recommendation for vitamin D during pregnancy is 200 IU/day [2], whereas the Institute of Medicine considers a daily intake of 600 IU/day [14]. In Mexico, the recommended intake in pregnancy is also 200 IU/day [15]. Most multivitamins on the market contain 200 or 400 IU of vitamin D. Recent clinical trials have shown that supplementation with >600 IU/day may decrease GDM [16]. The American College of Obstetrics and Gynecology considers doses of up to 1000 IU/day as safe during pregnancy [17].

After considering the evidence, the aim of this study was to describe maternal vitamin D status in healthy pregnant Mexican women and to evaluate the effect of frequently prescribed supplementation doses on longitudinal changes in 25-OH-D concentrations, adjusting for obesity, season, and other factors. 

## 2. Materials and Methods 

This study was part of the OBESO project at the Instituto Nacional de Perinatología (INPer) (Mexico City, Mexico). The OBESO project (Origen Bioquímico y Epigenético del Sobrepeso y la Obesidad) involves an institutional cohort of pregnant women and their children up to 2 years of age, which is aimed at studying the biochemical, clinical, lifestyle, and epigenetic determinants of obesity. The study was approved by the Ethics and Research Internal Review Board (Project No. 3300-11402-01-575-17). Participation was voluntary, and all women signed informed consent to agree to participation. Women were recruited at the Department of Maternal-Fetal Medicine in the first trimester of pregnancy. The sample was selected by convenience (January 2017–January 2020), according to inclusion criteria: healthy adult women, single pregnancy, without comorbidities (diabetes mellitus, renal or hepatic diseases, congenital malformations, autoimmune diseases, or uncontrolled thyroid disease), and not taking any medication that may affect endocrine metabolism (insulin, metformin, and/or corticosteroids). Women were eliminated from the analysis if they developed GDM and/or gestational hypertension or preeclampsia during pregnancy, or if data were incomplete. All women received routine prenatal care at INPer.

Women were assessed in the Nutrition Clinic during the three trimesters of pregnancy: T1 (11–13.6 weeks of gestation), T2 (18–22.6 weeks of gestation), and T3 (28–34.6 weeks of gestation). The gestational age at each visit was calculated according to the fetal ultrasound performed during the first trimester. Pregestational weight was reported by women in the first visit. Current weight was measured to the nearest ±0.1 kg, with women wearing light clothing and no shoes, using a calibrated digital scale (BMB-800, TANITA, Japan), and height was measured to the nearest 0.1 cm using a digital stadiometer (model 264, SECA, Hamburg, Germany), with the head placed in the proper position, according to the Frankfort plane. The pregestational body mass index (pBMI) (weight (kg)/height (m)^2^) was computed, and the women were classified according to the WHO criteria [18] as follows: normal weight (pBMI = 18.5 to 24.9), overweight (pBMI ≥ 25), or obese (pBMI ≥ 30). Demographic, clinical, and lifestyle data were obtained. Women were classified as nulliparous (had never delivered a baby) or as multiparous (had given birth at least once). The season of the year was registered at the time blood samples were taken and codified as spring/summer or autumn/winter. 

Supplementation was prescribed by obstetricians or other health professionals involved in prenatal care, and the decision was independent from the study. The women were asked about supplement use. The different brands of vitamin D supplements and multivitamins with vitamin D were obtained in each trimester. The daily vitamin D3 prescribed doses were calculated. The studied categories of supplementation were 0–250, >250–400, and >400 UI/day.

In each visit, the weight, season of the year, and supplementation were evaluated. Gestational weight gain during the third trimester was evaluated according to the Institute of Medicine guidelines, which considers gestational age and pregestational weight status. Women were classified as having insufficient, adequate, or excessive gestational weight gain in the third trimester [19].

A fasting blood sample was collected in the three trimesters of pregnancy (T1, T2, and T3) for different measurements from the OBESO cohort. Whole blood was centrifugated at 3500 rpm; one 500 μL serum aliquot was obtained for this analysis and frozen at −70 °C on the same day of blood collection. The quantification of 25-OH-D concentrations was performed by ELISA (quimioluminiscence; Architect Abott Diagnostics, Lake Forest, IL, USA), within 2–3 months of blood collection. The adjustment curve was created by duplication with 6 points. An acceptable coefficient of variation was considered as <5%. An insufficient status was considered when serum concentrations were <30 ng/mL, and a deficient status was considered when concentrations were <20 ng/mL [20].

### Statistical Analysis

Descriptive measures and frequencies were used to characterize data. To evaluate if data presented a parametric distribution, the Kolmogorov–Smirnov test was performed. Mean differences were analyzed with Student’s *t*-test and the one-way ANOVA test. Multivariate analysis included the development of general mixed-linear models to evaluate the effects of supplementation, doses, pregestational obesity, parity, and season on longitudinal serum maternal 25-OH-D concentrations (B, 95% CI). Statistical analysis was performed using STATA 14.0 (StataCorp, College Station, TX, USA). Statistical significance was considered for *p* < 0.05.

## 3. Results

By January 2020, 194 women had been included in the cohort and had completed their assessment during the third trimester. Women were not included in the analysis if they developed preeclampsia (*n* = 23), GDM (*n* = 7), or gestational hypertension (*n* = 4), and those with incomplete data were also excluded (*n* = 19). We present results from 141 women. 

The mean age was 29 years (range, 18 to 43 years). More than half of the women (63.9%, *n* = 90) had low educational levels. Of all the women, 23.4% (*n* = 33) had pregestational obesity and 34.8% (*n* = 49) were overweight. The maternal weight gain was 5.99 ± 3.30 kg in the third trimester. Excessive gestational weight gain was observed in 22% (*n* = 32) of the women; 39% (*n* = 56) showed adequate gestational weight gain. Table 1 shows the mean vitamin D concentrations during pregnancy by socio-demographic and clinical variables. 

Bivariate analysis showed higher 25-OH-D concentrations in supplemented than in non-supplemented women in the second and third trimesters. In the spring/summer season, women showed higher 25-OH-D concentrations in the third trimester. No other differences were observed.

In the first trimester of pregnancy, 51.4% of the women had vitamin D insufficiency and 37.3% had a deficient status. Only 45.8% of the women were supplemented with vitamin D at this time. Vitamin D supplementation was higher in the second and third trimesters (71.6% and 75.9%, respectively). In the second trimester, adequate vitamin D status was observed in 30.5% (*n* = 43) of the women, and 25.5% (*n* = 36) had vitamin D deficiency. In the third trimester, 20.5% (*n* = 29) of the women had a vitamin D deficient status. 

**Figure 1:**
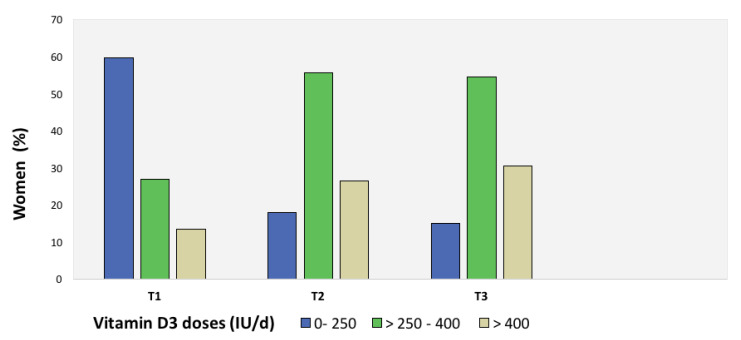
Doses of vitamin D3 supplementation during pregnancy in studied women.

**Figure 2:**
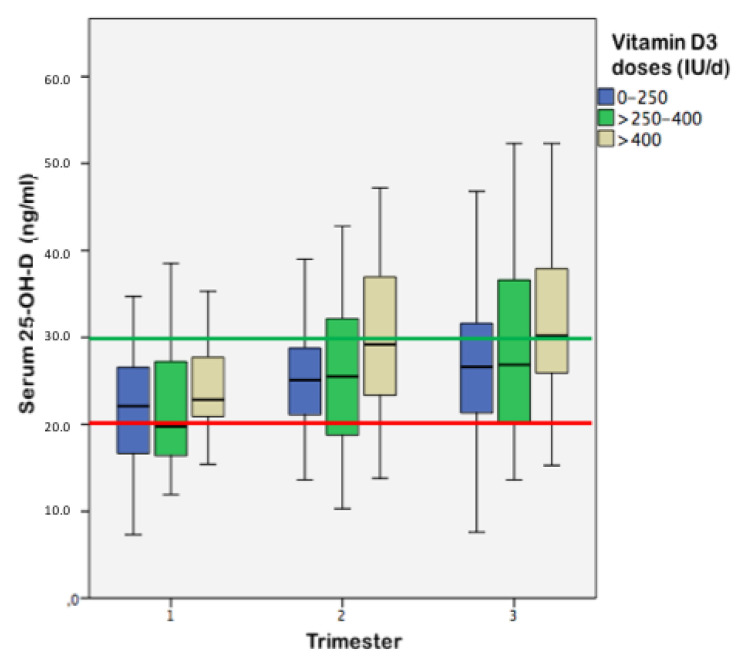
Serum vitamin D concentrations (25-OH-D) during pregnancy according to vitamin D3 supplementation. The green line represents the cut-off point for adequate vitamin D status (25-OH-D ≥ 30 ng/mL). The red line represents the cut-off point for vitamin D deficiency (25-OH-D < 20 ng/mL).

When analyzing doses, only 2.1% of the women in the first trimester received 600 IU/day or more of vitamin D3, increasing to 7.7% and 6.5% in the second and third trimesters, respectively. Most women (90%) received doses lower than 500 IU/day or no supplementation at all throughout pregnancy. The highest prescribed dose was 900 IU/day (*n* = 1). Figure 1 and Figure 2 show the frequency of vitamin D3 supplementation and doses used throughout pregnancy, and 25-OH-D concentrations according to supplementation doses, respectively. 

**Figure 3:**
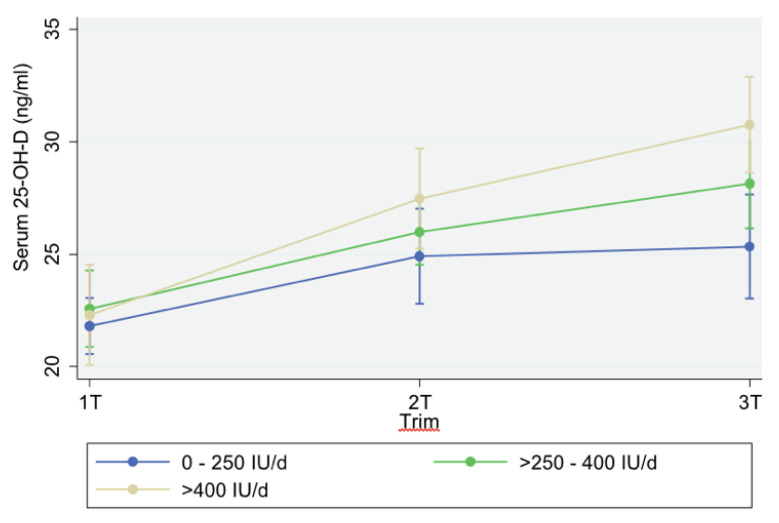
Mean vitamin D concentrations throughout pregnancy according to supplementation doses, season, pregestational weight status, parity, and age. Marginal means and 95% confidence intervals. T1, first trimester; T2, second trimester; T3, third trimester. General mixed-linear model, adjusted by season, pregestational weight status, parity, and age. *Significantly higher concentrations in T3 in the >400 IU/day group (*p* = 0.003).

The final general mixed-linear model showed that 25-OH-D concentrations increased significantly during pregnancy. The 25-OH-D concentrations were higher in the second and third trimesters compared to those in the first (*p* < 0.001; Table 2). A significant interaction was observed between the supplementation doses and trimester of pregnancy with increasing 25-OH-D concentrations. The highest increase in 25-OH-D concentrations during pregnancy was observed in women supplemented with more than 400 IU/day in the third trimester (+4.80 ng/mL, 95% CI: 1.72 to 8.11 ng/mL, *p* = 0.001), independently of season, pregestational weight status, parity, and age (Table 2). The mean 25-OH-D concentrations were significantly higher in women in the third trimester receiving >400 IU/day, adjusted for all other factors (Figure 3). 

The season of the year was also a significant determinant of 25-OH-D concentrations during pregnancy. An overall significant change of −1.85 ng/mL (95% CI: −2.99 to −0.72 ng/mL, *p* = 0.001) was observed when samples were taken in autumn/winter compared to when they were taken in spring/summer. A negative effect on 25-OH-D concentrations was also observed in women with pregestational obesity (−2.42, 95% CI: −5.29 to 0.43 ng/mL, *p* = 0.09); however, it was not statistically significant (Table 2). Gestational weight gain was not associated with 25-OH-D concentrations or vitamin D status, so it was not included in the final model.

## 4. Discussion

This is one of the few studies prospectively assessing maternal 25-OH-D concentrations considering the doses of vitamin D3 supplementation in healthy pregnant women, while adjusting for other determinants of vitamin D status. We observed an increase in 25-OH-D concentrations throughout pregnancy. The vitamin D concentrations in the second and third trimesters were significantly higher than the first trimester concentrations. Other studies reported this increase in 25-OH-D concentrations with gestational age [21,22].

We observed a very high prevalence of vitamin D deficiency and insufficiency, particularly in the first trimester, where 51% of the women presented with insufficiency and 37%, with deficiency. The estimated prevalence of vitamin D deficiency (<20 ng/mL) in North America (USA and Canada) was reported to be 42–72% [6]. In a longitudinal study in Sweden, 37% of women in the first trimester had 25-OH-D concentrations of <20 ng/mL [22], whereas in a Canadian cohort, 23% of women showed deficiency at this cut-off point [23]. In a previous cross-sectional study in Mexico, 61% of women in the third trimester and 98% of their newborns had vitamin D deficiency (<20 ng/mL) [9]. The differences with this specific study may be due to the women included in our study being healthy. INPer is a third-level hospital and receives high-risk women, so the usual clinical care may have included more interventions (including supplementation) than that provided in other general hospitals in Mexico. In our study, 75% of women received vitamin D3 supplementation in the third trimester. No previous longitudinal data have been reported in pregnant Mexican women.

Vitamin D deficiency and insufficiency have been associated with different perinatal adverse outcomes. In a review of observational studies (87 studies, *n* = 29,902 women), low 25-OH-D concentrations were associated with a higher risk of GDM (Odds Ratio-OR: 1.85; 95% CI: 1.47 to 2.32) [10]. In another review, a higher risk of preterm birth was also reported (OR: 1.29; 95% CI: 1.16 to 1.45) [11]. A higher risk of preeclampsia was observed in women with 25-OH-D <30 ng/mL (OR: 1.79; 95% CI: 1.25 to 2.58); however, when adjusting for confounding variables, the association was lost [24]. All this evidence supports the promotion of an adequate vitamin D status during pregnancy as a priority during prenatal care.

The main source of vitamin D is sun exposure; however, diet and supplementation also provide important quantities. Many risk factors for vitamin D deficiency have been described, including having dark skin, low sun exposure (winter, Nordic countries), pollution, and obesity, among others [4,24]. The dietary reference intakes for vitamin D during pregnancy vary among different organizations and countries [14,15]. Due to inconsistencies regarding the evidence from studies evaluating vitamin D supplementation and lower risks of adverse perinatal outcomes, the routine supplementation of this vitamin during pregnancy is not recommended at this time [2,12], but controversy exists regarding this topic. Studies of vitamin D supplementation to reduce GDM, preterm birth, or preeclampsia risk failed to show strong effects due to the high heterogeneity of the interventions (regarding doses and the time of supplementation, among others). In a recent Cochrane review, a reduction in the risk of GDM was reported in women supplemented with more than 600 IU/day compared to that for women receiving lower doses (Relative Risk-RR: 0.54; 95% CI: 0.34 to 0.86; five randomized clinical trial (RCT); *n* = 1846 women). In studies evaluating the effect on the risk of preeclampsia, preterm birth, or low birth weight, a minimal or no difference was observed in the risk of these outcomes, with 600 IU/day or more of vitamin D. When evaluating supplementation with much higher doses (>4000 vs. 4000 IU/day or lower), there was no effect on GDM risk or other complications [16]. 

In another meta-analysis of 24 RCT (*n* = 5405), supplementation with >400 IU/day of vitamin D3 was associated with a lower risk of having a small-for-gestational-age newborn (RR: 0.72; 0.52 to 0.99) and with lower fetal and neonatal mortality. Doses of 2000 IU/day or higher did not exert these benefits [25].

Even though 75% of the women in our study received supplementation in the third trimester, 20% still showed vitamin D deficiency. In general, the doses prescribed were low. Doses of 400 IU/day or lower appeared to be insufficient for achieving adequate vitamin D status in these women (Figure 3). Only 10% of the women received doses of 500 IU/day or higher. Considering the current dietary intake recommendations from the WHO, this practice appears to be common around the world. The multivariate analysis in our study showed that the most important factor in the increasing 25-OH-D concentrations in the third trimester was the supplementation with >400 IU/day, independently of other determinants (season, obesity, parity, and age). In a mother–offspring cohort in Canada (*n* = 1753), vitamin D supplementation (yes/no) was the strongest predictor of vitamin D tracking during pregnancy [13]. In a recent longitudinal study from Sweden, receiving a multivitamin with vitamin D (in the last 14 days) was also an important determinant of plasma 25-OH-D [22]. In these studies, doses were not considered in the analysis. 

In a study in India (medium socioeconomic status), prenatal vitamin D supplementation with 400 IU/day was not effective in preventing low umbilical cord 25-OH-D concentrations; it was reported that 97% of neonates were deficient [26]. In a randomized clinical trial from Iran, vitamin D supplementation with 1000 IU/day was compared to that with 2000 IU/day. In both groups, 25-OH-D concentrations increased, but a significantly higher increase was observed in the 2000 IU/day group [27]. 

The upper limit for dietary intake in pregnancy according to the Endocrine Society and Institute of Medicine is 4000 IU/day [14,20]. In a RCT in women in Bangladesh, the effect of weekly supplementation with 35,000 IU/week of vitamin D3 (similar to 5000 IU/day) on cord blood 25-OH-D concentrations was evaluated. Almost all the newborns (95%) and 100% of the mothers in the intervention group achieved 25-OH-D concentrations >20 ng/mL. No hypercalcemia or adverse effects were observed [28]. In another RCT where doses of 400 IU/day were compared to higher doses (1400, 2400, and 3400 IU/day), 25-OH-D concentrations increased significantly from 20 to 36 weeks of gestation in a dose-dependent manner. At 36 weeks, only with doses of 1400 IU/day did 97.5% of women present with an adequate status. No adverse effects or signs of vitamin D toxicity were reported [29]. 

The season of the year, mainly winter and autumn, is related with lower 25-OH-D concentrations. Higher concentrations have been observed in summer (mean: 27.84 ng/mL) than in winter (mean: 20.08 ng/mL) (*p* < 0.001) in pregnant Swedish women [22]. A significant increase of 3.09 ng/mL in 25-OH-D concentrations was reported in spring, summer, or autumn compared to winter concentrations (*p* < 0.001) [23]. In general, most of the women included in our study lived in Mexico City and near urban areas, where most people do not receive frequent sun exposure to promote endogenous vitamin D synthesis. This may be related to higher pollution levels and less exposure to UV light, as well as the higher frequency of indoor activities. Our data showed that, as in other countries such as Canada and Sweden, vitamin D concentrations during pregnancy are lower in Mexico during the autumn and winter seasons. 

In general, obesity is associated with a higher risk of vitamin D deficiency. This may be explained by apparently less sun exposure in individuals with obesity and excess body fat retaining vitamin D metabolites, and because cholecalciferol produced through the skin or acquired through the diet is partially sequestered by body fat [30]. Vitamin D receptors are widely expressed in adipose cells, which have the capacity to activate vitamin D with the 1-alpha-hydroxilase enzyme. In vitro experiments demonstrated that vitamin D plays a key role in adipocyte metabolism by inhibiting the differentiation of pre-adipocytes and by suppressing a number of transcriptional regulators and functional proteins [31].

Women who were obese before pregnancy (pBMI > 29.9) had slightly lower 25-OH-D concentrations, but this effect was not significant (*p* = 0.09). The effect of higher pBMI in decreasing 25-OH-D concentrations during pregnancy was reported in some studies; however, the findings have not been consistent [13,21].

Considering the high prevalence of vitamin D deficiency in many countries, it seems appropriate to revise and update dietary intake recommendations during pregnancy around the world, including in Mexico. It is relevant to include vitamin D status assessment in prenatal care to individualize supplementation schemes. Higher doses (>400 IU/day) may be required for pregnant women, mainly in autumn and winter seasons, and in women with obesity. More studies are needed, particularly randomized clinical trials, to evaluate higher doses of supplementation in healthy pregnant women and in women with different baseline risk factors for deficiency. 

The strengths of this study include its longitudinal design and the close monitoring of women during pregnancy, as well as the detailed information about individual supplementation schemes (with doses) and other determinants of vitamin D status. Another strength is the performed analysis, which allowed us to adjust for many confounding factors. Some limitations are the relatively small sample size and the fact that the studied women were selected with rigorous criteria. For that reason, our results may not be generalizable to all women. Another limitation was that we were not able to assess vitamin D intake from dietary sources. However, oral vitamin D intake in pregnancy is largely represented by supplements. Ideally, other biochemical markers that are directly associated with vitamin D status (parathyroid hormone, vitamin D-binding protein, and calcium) could have been measured to better characterize the women’s health status. Finally, the observational design of this study did not allow for the control of vitamin D3 supplementation.

## 5. Conclusions

In this group of Mexican pregnant women, the prevalence of vitamin D deficiency and insufficiency was high. Vitamin D3 supplementation was not a routine practice in all trimesters of pregnancy. The increase in vitamin D concentrations observed with gestational age was the highest when women were supplemented with >400 IU/day. The most common recommended doses (250–400 IU/day) were not enough to achieve adequate vitamin D status. In the autumn and winter seasons, maternal 25-OH-D was lower during pregnancy. 

## Figures and Tables

**Table 1:** Maternal 25-OH-D concentrations during pregnancy according to sociodemographic and clinical data.

Variable	All Women (%, *n*)	25-OH-D Concentrations (ng/mL) (Mean ± SD)		
		First Trimester T1	Second Trimester T2	Third Trimester T3
Maternal Age (years) (Mean ± SD)	29.4 ± 5.162			
Education Level				
<High School	63.9% (90)	22.58 ± 6.29_a_	26.25 ± 8.74_a_	28.07 ± 9.34_a_
High School/Professional	36.2% (51)	20.95 ± 6.84	25.60 ± 8.87	28.04 ± 10.66
Occupation				
Homemaker	65.2% (92)	22.16 ± 5.99_a_	26.16 ± 8.87_a_	28.33 ± 9.64_a_
Other	34.8% (49)	21.67 ± 7.46	25.74 ± 8.65	27.53 ± 10.16
Parity				
Nulliparous	53.2% (75)	21.69 ± 5.78_a_	26.26 ± 8.64_a_	29.26 ± 9.32_a_
Multiparous	46.8% (66)	22.33 ± 7.29	25.51 ± 8.94	26.71 ± 10.20
Pregestational BMI (kg/m^2^) (Mean ± SD)	27.99 ± 4.888			
Pregestational Weight Status				
Normal	41.8% (59)	22.86 ± 6.50_b_	27.96 ± 8.92_b_	27.92 ± 9.38_b_
Overweight	34.7% (49)	22.00 ± 7.00	24.74 ± 9.11	29.10 ± 10.97
Obese	23.4% (33)	20.42 ± 5.62	24.42 ± 7.42	26.75 ± 8.71
Gestational Weight Gain (kg) in T3 (Mean ± SD)	5.99 ± 3.30			
Gestational Weight Gain (T3)				
Low	37.6% (53)	22.71 ± 6.69_b_	26.40 ± 8.47_b_	27.92 ± 9.46_b_
Adequate	32.7% (56)	21.83 ± 6.84	25.90 ± 8.21	28.32 ± 10.18
Excessive	22.7% (32)	21.09 ± 5.65	25.56 ± 10.31	27.82 ± 9.96
Season				
Spring/Summer	40.4% (84)	22.68 ± 6.50_a_	27.07 ± 8. 66_a_	29.93 ± 9.44_a_ **
Autumn/Winter	59.5% (57)	20.97 ± 6.46	24.45 ± 8.76	25.25 ± 9.73
Vitamin D3 Supplementation (Anytime)				
Not Supplemented	10.6% (15)	18.48 ± 6.48_a_	20.65 ± 8.95_a_ *	20.96 ± 7.24_a_ **
Supplemented	89.4% (126)	22.41 ± 6.58	26.65 ± 8.60	28.91 ± 9.74

_a_ Student’s *t*-test; _b_ One-way ANOVA; * *p* ≤ 0.05; ** *p* ≤ 0.01.

**Table 2:** Effect of vitamin D3 supplementation and doses of supplementation on maternal 25-OH-D concentrations (ng/mL) throughout pregnancy, adjusted for other factors.

Variable	Coefficient	Standard Error	95% CI		*p*-Value
Trimester of Pregnancy					
T2	**3.100**	**0.86**	**1.41**	**4.78**	**<0.001**
T3	**3.53**	**1.06**	**1.43**	**5.63**	**0.001**
Vitamin D3 supplementation Doses (IU/day)					
>250–400	0.76	0.93	−1.07	2.60	0.414
>400	0.49	1.21	−1.88	2.86	0.685
Trimester × supplementation dose					
Second trimester × 250–400 IU/day	0.31	1.06	−1.76	2.39	0.765
Second trimester × >400 IU/day	2.06	1.61	−1.10	5.23	0.201
Third trimester × >250–400 IU/day	2.04	1.58	−1.06	5.14	0.197
**Third trimester × >400 IU/day**	**4.92**	**1.62**	**1.72**	**8.11**	**0.003**
Pregestational weight status					
Overweight (pBMI > 24.9)	−0.61	1.48	−3.52	2.30	0.680
Obese (pBMI > 29.9)	−2.42	1.46	−5.29	0.43	0.09
Season of the year					
**Autumn/winter**	**−1.85**	**0.57**	**−2.99**	**−0.72**	**0.001**
Parity					
Multiparous	−0.20	1.35	−2.85	2.44	0.880
Age (years)	−0.59	0.13	−0.32	0.20	0.662

General mixed linear model. Reference groups: trimester of pregnancy—first trimester; vitamin D3 supplementation doses—<250 IU/d; pregestational weight status—normal-weight women; season of the year—spring–summer; parity—nulliparous women. Variables in bold were statistically significant in the model.
